# Wearable Technology and Sensors for Healthcare and Wellbeing

**DOI:** 10.3390/s26082385

**Published:** 2026-04-13

**Authors:** Salvatore Tedesco, Dimitrios-Sokratis Komaris

**Affiliations:** 1 School of Computer Science & IT, University College Cork, Western Gateway Building, Western Road, T12 XF62 Cork, Ireland; 2 Tyndall National Institute, University College Cork, Lee Maltings Complex, Dyke Parade, T12 R5CP Cork, Ireland; 3 Information Technologies Institute, Centre of Research & Technology—Hellas, 57001 Thessaloniki, Greece

## 1. Introduction

Wearable technology has undergone explosive growth over recent years, driven by sweeping advances in information and communications technology and shaped by fundamental shifts in demography, lifestyle, and the broader environment [1,2]. What began as personal tracking devices [3,4]—counting steps and monitoring heart rate—has rapidly matured into a sophisticated ecosystem of sensors and systems capable of supporting personalised healthcare, sports analytics, rehabilitation, and chronic disease management [5]. The continuous proliferation of connected wearable devices has, in turn, generated vast quantities of heterogeneous data, demanding commensurate advances in machine learning and artificial intelligence to enable real-time pattern recognition, meaningful inference, and actionable feedback [6]. In this way, wearables are no longer passive recorders of physiological data but active participants in a broader digital health ecosystem, with the potential to transform the delivery of care and the monitoring of wellbeing at scale.

This Special Issue [7], “Wearable Technology and Sensors for Healthcare and Wellbeing”, was conceived to advance the state of the art by soliciting research contributions at the intersection of novel wearable hardware, sensor systems, signal processing, and machine learning, with particular attention to applications in human motion analysis, telerehabilitation, geriatric care, Parkinson’s disease, physiological monitoring, emotion recognition, sports analytics, and injury prevention. The response from the community was encouraging, and this editorial provides an overview of the contributions accepted for publication and identifies the major themes and research gaps they highlight.

## 2. Overview of Contributions

The thirteen contributions accepted for this Special Issue collectively span a wide arc of wearable sensor research, from novel hardware prototypes and robotic assistive devices to signal processing algorithms, deep learning architectures, and large-scale observational studies.

With regard to the hardware and device layer, two contributions push the boundaries of what wearable and wearable-adjacent systems can do.

The STELO device (Contribution 1 [8]) is a modular gait-assistive exoskeleton designed for individuals with acquired brain injury (ABI). Unlike conventional rigid, bilateral exoskeletons, STELO can be externally configured with joint modules to cater to the diverse impairment profiles of each patient. Evaluated across 14 ABI-diagnosed participants over three sessions, the device demonstrated a strong safety profile, with progressive reductions in the need for adjustment assistance and satisfactory ratings of comfort and usability from both participants and therapists.

In a complementary vein, the study by Leharanger et al. (Contribution 2 [9]) moves the hardware discussion into the domain of extended reality, investigating the familiarisation of eleven individuals with severe Autism Spectrum Disorder (ASD) with the HoloLens 2 Mixed Reality (MR) headset. Using eye tracking as a quantitative learning indicator alongside execution speed, the study finds that 81.81% of participants with ASD successfully familiarised themselves with the MR environment and that their visual activity patterns became comparable to those of neurotypical individuals upon successful familiarisation.

Together, these two contributions illustrate that hardware innovation in wearables is as much about adaptability and accessibility as it is about technical performance.

Sensor validation and the establishment of measurement reliability form another important cluster of work in this Special Issue.

A prototype sport watch (Polar Electro Oy) is rigorously evaluated against overnight polysomnography—the gold standard for sleep staging—in 36 young adults under both resting and post-exercise conditions (Contribution 3 [10]). The prototype achieves accuracy and agreement levels comparable to or better than existing consumer wrist-worn devices, with no significant differences between exercise and non-exercise nights, providing a solid empirical basis for its use in health-monitoring applications.

This question of what wearable devices can reliably measure is also at the heart of the large-scale observational study leveraging data from nearly one million days and nights logged by 11,914 WHOOP subscribers (Contribution 4 [11]). Here, the focus shifts from device validation to the real-world consequences of sustained engagement: higher wear frequency is associated with lower resting heart rate, higher heart rate variability, longer and more consistent sleep, and greater physical activity, with mediation analyses suggesting that improved sleep duration is a key pathway to better cardiovascular outcomes. The study is notable for its scale and its use of both between- and within-person modelling, offering compelling real-world evidence that consistent wearable use may itself be health-promoting.

The largest group of contributions addresses the computational challenge of extracting clinically meaningful information from raw sensor signals, and here the diversity of approaches reflects the diversity of the problems being solved.

For physiological monitoring, two papers focus on optical sensing via photoplethysmography (PPG). A personalised multiclass classification model using PPG features from both the brachial and digital arteries is developed to detect blood pressure variations under physical and cognitive workload, achieving 95.1% aggregated accuracy through a transfer learning strategy that combines target and source subject data (Contribution 5 [12]).

Taking multi-channel optical sensing further, a quaternion-valued signal processing framework exploits the hidden inter-channel relationships in four-channel PPGs to enable non-invasive blood glucose estimation, with principal component analysis applied for denoising (Contribution 6 [13]); the resulting random forest model outperforms both conventional and existing quaternion-based feature sets across two independent datasets. These two contributions, read together, illustrate the growing sophistication of wearable optical sensing and the gains that can be made by moving beyond single-channel, population-level approaches.

The theme of intelligent signal interpretation continues in the domain of human activity and motion analysis. A deep neural network combining convolutional and long short-term memory architectures is proposed for the concurrent identification of multiple health-related behaviours—smoking, exercising, eating, and medication taking—from wrist-worn accelerometers, achieving a macro-F1 score of at least 85.1% in a leave-one-subject-out evaluation of 60 participants (Contribution 7 [14]).

This work is complemented by a study employing a self-attention mechanism within a temporal deep learning model for the continuous estimation of ankle and knee joint angle trajectories across varied locomotion modes, a capability with direct implications for the adaptive control of lower limb prostheses and exoskeletons (Contribution 8 [15]). Transfer learning is shown in this latter work to meaningfully reduce model error, underscoring the value of data diversity. Together, these papers point to the convergence of deep learning and wearable sensing as a particularly productive frontier, where the complexity of real-world movement can increasingly be captured and decoded.

Beyond the recognition of physical activity, several contributions explore what wearable physiological data can reveal about mental and psychological health—a dimension of wearables research that is gaining significant momentum.

The feasibility of identifying clinical depression from multimodal wristband data—pulse wave, skin conductance, and triaxial acceleration—collected over six hours of daily activity is demonstrated in a study of 58 clinically diagnosed individuals and 58 matched controls, with a Random Forest classifier achieving up to 90.0% accuracy on 6 h segments (Contribution 9 [16]).

Taking a longer temporal view, a contribution drawing on the LifeChamps cancer care dataset combines wearable sensor data with continuous self-reported outcomes to build dynamic, personalised risk models for vulnerability and anxiety in older adult cancer survivors, using process mining to capture how risk factors evolve over time rather than treating them as static (Contribution 10 [17]).

These two studies, alongside the MR familiarisation work in ASD populations [9], collectively reflect a broader shift in the field toward wearable applications that address mental health, neurodevelopmental conditions, and the psychosocial dimensions of chronic illness.

The Special Issue also makes a meaningful contribution to sports science and injury prevention. An inertial measurement unit system combined with force-velocity profiling is applied in a feasibility study examining associations between sprint biomechanics and hamstring strain injury in 23 amateur sprinters followed over a year, identifying increased anterior pelvic tilt as a cross-sectional differentiator and suggesting that a velocity-oriented sprint profile may be prospectively linked to injury risk (Contribution 11 [18]).

Addressing the broader integration of wearable technology into sports psychology, a critical review of 3D-printed wearable sensors examines both their technical potential and their real-world applicability in athlete health monitoring, identifying a significant gap in the evaluation of sensor-based interventions on coaching decision-making and psychological adaptation (Contribution 12 [19]). These contributions highlight that wearable technology in sport is evolving well beyond performance tracking, toward tools that can inform injury prevention strategies and psychological support programmes.

Finally, one contribution steps back from physiological and behavioural signals to address the haptic sensing and design challenges that underpin remote medical examination (Contribution 13 [20]). Through the systematic characterisation of hand motions and interaction forces during thyroid palpation—drawing on video recordings, expert interviews, and force measurements from both medical professionals and non-medical participants—five primary palpation techniques and their associated multi-dimensional force profiles are identified. These findings provide directly actionable design guidelines for sensorized gloves and robotic systems capable of transmitting haptic information for remote diagnosis, with particular relevance to settings where specialist practitioners are scarce. In doing so, this work extends the concept of wearable sensing beyond the patient and into the hands of the clinician, opening a new dimension in the broader wearable healthcare ecosystem.

## 3. Emerging Themes and Cross-Cutting Observations

Taken together, the contributions to this Special Issue highlight several cross-cutting themes that reflect the current trajectory and challenges of the field.

*Machine learning as a unifying enabler*

Across application domains—from gait rehabilitation and sleep staging to depression detection and blood glucose estimation—machine learning and deep learning methods serve as a common methodological thread [12,13,14,15,16,17]. Convolutional and recurrent neural networks, attention mechanisms, random forest classifiers, and transfer learning all feature prominently, underlining the central role of data-driven approaches in extracting clinically meaningful information from wearable sensor data. The shift from hand-crafted features toward end-to-end learning pipelines, while promising, also raises important questions about model interpretability, generalisability, and the data requirements for robust training.

*Personalisation and user-centred design*

Several contributions emphasise the importance of personalisation, whether in the form of patient-specific classification models, modular hardware configurations, or dynamic risk models that account for the time-varying nature of individual health states [8,12,17]. This reflects a broader recognition that population-level models, while useful as baselines, often fail to capture the heterogeneity of real-world users [21]. Personalisation comes with its own challenges, however, including the need for sufficient individual-level training data and mechanisms for efficient model updating as a user’s condition evolves.

*Broadening clinical and sporting application domains*

The scope of this Special Issue spans acute rehabilitation settings, chronic disease management, oncology, mental health, and elite sport, demonstrating that wearable sensor technology is permeating an increasingly wide range of healthcare contexts. Notably, contributions addressing depression recognition [16], cancer survivor monitoring [17], and ASD familiarisation with MR technology [9] reflect a growing interest in the application of wearables to populations and conditions that have historically been underserved by digital health innovation.

*Hardware innovation alongside algorithmic progress*

While the majority of contributions focus on algorithmic and analytical advances applied to commercially available or prototype sensors, several papers address hardware innovation directly. The STELO modular exoskeleton [8], 3D-printed sensor systems for sports psychology [19], and the characterisation of haptic forces for palpation sensor design [20] each highlight that meaningful progress at the hardware level remains essential for expanding the capabilities and applicability of wearable systems.

*Ecological validity and real-world deployment*

A recurring challenge across contributions is the translation from controlled laboratory validation to real-world deployment. Several studies acknowledge limitations arising from small sample sizes, homogeneous participant demographics, or highly controlled experimental conditions [3,4]. The large-scale WHOOP study [11] aims to overcome those challenges and offers insights into the population-level effects of wearable engagement based on observational real-world data. As the field matures, prospective, longitudinal, and multi-site studies will be essential to establish the clinical and practical utility of wearable sensor systems.

## 4. Research Gaps and Future Directions

Several important research gaps and future directions emerge from the body of work gathered in this Special Issue.

Firstly, there is a pressing need for standardised benchmarking frameworks and publicly available datasets to enable meaningful comparison across methods and laboratories. The diversity of hardware platforms, preprocessing pipelines, participant populations, and evaluation metrics currently makes it difficult to draw definitive conclusions about the relative merits of competing approaches.

Secondly, while machine learning approaches have demonstrated impressive performance in constrained settings, questions of robustness and generalisability to diverse populations—including older adults, individuals with disabilities, and clinical populations—remain largely open [22]. Contributions in this Special Issue represent initial steps in this direction, but substantial further work is required to develop models that perform reliably across the full spectrum of potential users.

The integration of multimodal data streams—combining physiological signals, motion data, and self-reported outcomes, as exemplified by the cancer survivor monitoring contribution—offers a particularly fertile avenue for future research [23]. Multimodal fusion has the potential to provide a richer and more nuanced picture of an individual’s health state than any single sensing modality alone.

The ethical, regulatory, and data governance dimensions of wearable health monitoring—including issues of data privacy, informed consent, and algorithmic accountability—warrant greater attention from the research community [24]. As wearable systems move closer to clinical deployment, engagement with regulators and ethicists will be essential.

Finally, the potential of edge computing and on-device inference to enable truly real-time, privacy-preserving wearable analytics—as envisioned in the Special Issue call—remains an important direction for both hardware and software research [25]. Reducing reliance on cloud infrastructure will be essential for deploying wearable AI systems in low-connectivity environments and for individuals with the greatest need.

## 5. Conclusions

The thirteen contributions gathered in this Special Issue collectively advance the science and application of wearable technology for healthcare and wellbeing. From modular rehabilitation robots and deep learning models for gait estimation to depression recognition systems and large-scale observational studies of wearable engagement, they reflect the remarkable breadth and ambition of contemporary research in this space. They also underscore the need for continued methodological rigour, inclusive study designs, and closer collaboration between engineers, clinicians, and end-users.

We are grateful to all authors, reviewers, and the editorial team for their efforts in bringing this Special Issue to fruition, and we look forward to the continued development of the field.
